# The efficacy of surgical interventions for trigeminal neuralgia in multiple sclerosis: A systematic review

**DOI:** 10.1007/s10143-026-04494-5

**Published:** 2026-10-07

**Authors:** Anish Kalyana, Daniel Coles, Sameer Khalil, Mohammad Mahmud, Roshni Beeharry, Sanjay Cheema, Anouk Borg, Tim M Young

**Affiliations:** 1 Queen Square Institute of Neurology, University College London, London, UK; 2 National Hospital for Neurology and Neurosurgery, London, UK

**Keywords:** Multiple sclerosis, Secondary trigeminal neuralgia, Microvascular decompression, Percutaneous ablative procedures, Gamma knife radiosurgery, Trigeminal neuralgia

## Abstract

Trigeminal neuralgia affects approximately 3% of patients with multiple sclerosis – a far higher prevalence than in the general population. The associated pain often proves refractory to medication, prompting consideration of neurosurgical intervention. The variable efficacy, tolerability, and durability of available procedures, compounded by factors such as multiple sclerosis subtype and neuroimaging findings, require evidence to guide personalised surgical decision-making. This systematic review evaluates surgical interventions for trigeminal neuralgia in multiple sclerosis, analysing how patient and disease-specific variables influence outcomes. A systematic review with narrative synthesis was performed. Following PRISMA guidelines, Embase, PubMed, and Scopus were searched (January 2000 - April 2026). Studies assessing pain relief from surgical procedures for trigeminal neuralgia in multiple sclerosis using the Barrow Neurological Institute Pain Scale were included. Risk of bias was assessed using the Newcastle-Ottawa Scale. Thirty-two studies, encompassing 1617 patients, met the inclusion criteria. Microvascular decompression in cases with demonstrable neurovascular conflict provided durable outcomes offset by higher risks of serious complications (including one fatality), despite inherent diagnostic uncertainty regarding whether pain was specifically driven by multiple sclerosis. Partial sensory rhizotomy displayed favourable pain relief (initial success range: 95.3–100%) and durability (recurrence ranging from 20.1 to 79 months). Whilst some studies noted a lack of major complications, some cases of severe trigeminal dysfunction including anaesthesia dolorosa were reported (n = 3; 7.5%). Percutaneous ablative procedures (initial success ranging from 58 to 100%) and gamma knife radiosurgery (initial success ranging from 52.7 to 97.3%) achieved comparable pain control rates to classical trigeminal neuralgia cohorts. They also featured a mild complication profile, though durability was suboptimal (recurrence ranging from 7.4 to 74.5 months for gamma knife radiosurgery and 8–60 months for percutaneous procedures) and repeat procedures were typically tolerable. Differing gamma knife radiosurgery dosages (</>80 Gy) were not associated with significant differences in pain control. Preliminary outcomes of arachnoid-trigeminal nerve release from a 2025 study highlighted promising results with 75% of patients achieving Barrow Neurological Institute Pain Scale I at final follow-up. Gamma knife radiosurgery and percutaneous ablative procedures represent well-tolerated surgical options with comparable outcomes to primary trigeminal neuralgia cohorts regardless of plaque burden. Partial sensory rhizotomy demonstrated notable efficacy and durability in refractory multiple sclerosis-associated trigeminal neuralgia, particularly in the absence of neurovascular conflict, and warrants earlier consideration in the surgical pathway. Microvascular decompression may offer long-term durability where neurovascular conflict is demonstrated. However, its higher procedural risk and the diagnostic uncertainty inherent in multiple sclerosis-associated trigeminal neuralgia cases with concurrent neurovascular conflict necessitate cautious patient selection. High-resolution preoperative imaging is therefore recommended to guide personalised modality selection. Prospective studies are needed to further validate surgical decision-making and establish standardised outcome reporting in this population.

**Supplementary Information:**

The online version contains supplementary material available at https://doi.org/10.1007/s10143-026-04494-5.

## Introduction

### Background

Trigeminal neuralgia (TN) is characterised by unilateral, severe, brief, electric shock–like pain in the distribution of the trigeminal nerve, with an estimated lifetime prevalence of 0.3% in the general population [1]. TN may be primary or secondary to underlying pathology, most commonly multiple sclerosis (MS). TN occurs more frequently in MS, affecting approximately 1.9–4.9% of patients [2]. When TN is associated with MS and accompanied by at least one demyelinating plaque at the trigeminal root entry zone or within the pons, it is classified as MS-related trigeminal neuralgia (MS-TN) [3]. Throughout this manuscript, the term “primary TN” is used to refer to cohorts lacking a secondary cause and therefore includes idiopathic TN. Classical TN refers to cases where the cause is presumed to be vascular compression of the trigeminal nerve.

Clinically, MS-TN resembles classical and idiopathic TN, with sudden, unilateral, electric shock-like pain [4], though it presents at a younger age, is more often bilateral (in approximately 18% of cases), and features fewer triggers and shorter, less predictable pain-free intervals [4, 5]. Secondary TN accounts for up to 15% of all TN cases, with MS conferring an approximately 20-fold increased relative risk [4]. These features support MS-TN as a distinct clinical entity with the absence of formal, evidence-based management guidelines representing a significant clinical gap.

TN significantly impairs quality of life, with patients often avoiding daily activities for fear of triggering attacks [6]. In MS-TN, pharmacological therapies are frequently poorly tolerated, have limited evidence of effectiveness, and are associated with earlier drug resistance and need for surgical intervention compared to classical TN [4, 7, 8].

Classical TN typically arises from neurovascular conflict (NVC) causing focal demyelination at the root entry zone, whereas MS-TN is primarily driven by pontine demyelinating plaques, though NVC may coexist, potentially producing a synergistic “double-crush” mechanism [4, 9, 10]. MS itself is immune-mediated, most commonly relapsing-remitting (RRMS) with possible progression to secondary progressive MS (SPMS), while primary progressive MS (PPMS) shows steady decline from onset [11, 12].

### Surgical interventions

Surgical treatment of MS-TN aims either to relieve NVC or to disrupt nociceptive transmission. Microvascular decompression (MVD) separates contacting vessels from the trigeminal nerve and is the preferred treatment for classical TN with NVC, although benefit has also been reported in selected MS-TN cases [13–15]. In microsurgical partial selective rhizotomy (PSR), the nerve sensory root is selectively sectioned according to the distribution of pain [16]. PSR is similar to internal neurolysis (INL) with both procedures requiring a craniotomy and often undertaken when no NVC is found – however, PSR involves a selective dissection of part of the trigeminal sensory root whereas INL involves longitudinal division of the trigeminal nerve (nerve combing) into fascicles [14, 16]. A novel microsurgical intervention for refractory trigeminal neuralgia in the absence of NVC has been described recently in which the trigeminal nerve was released from tethering arachnoid (detethering), achieved by endoscopic-guided incision along the cisternal length of the nerve [17].

Ablative procedures are understood to interrupt pain pathways via different mechanisms. Percutaneous balloon compression (PBC) selectively injures large, myelinated fibres at the trigeminal ganglion; glycerol rhizotomy (GR) induces osmotic axonal injury; radiofrequency lesioning (RFL) produces thermal neurolysis and gamma knife radiosurgery (GKR) delivers stereotactic radiation to the trigeminal root [18–20]. While these techniques can provide durable pain relief in classical TN [20–22], outcomes in MS-TN are generally less favourable, with higher recurrence rates, limited data on repeat procedures, and no consensus regarding optimal surgical strategy [23–25].

## Rationale

Previous reviews - including Spina et al. [26] and Ali et al. [25] focussed on single interventions like GKR, whilst broader studies such as SohrabiAsl et al. [27], and Texakalidis et al. [28], , examined only percutaneous procedures, without offering side-by-side comparisons of invasive and minimally invasive techniques. There remains a lack of cohesive analyses that consider patient and pathological variables affecting treatment efficacy and safety. To date, no review has directly addressed all these aspects.

## Aims

This current systematic review aimed to provide an up-to-date analysis of current neurosurgical treatments for MS-TN, focusing on pain relief, side effects, and how outcomes vary by MS subtype. It also considered key factors shaping treatment pathways, such as NVC, demyelinating plaques, and radiation dosages for gamma knife treatments in MS-TN.

## Materials and methods

### Search strategy

**Fig. 1:**
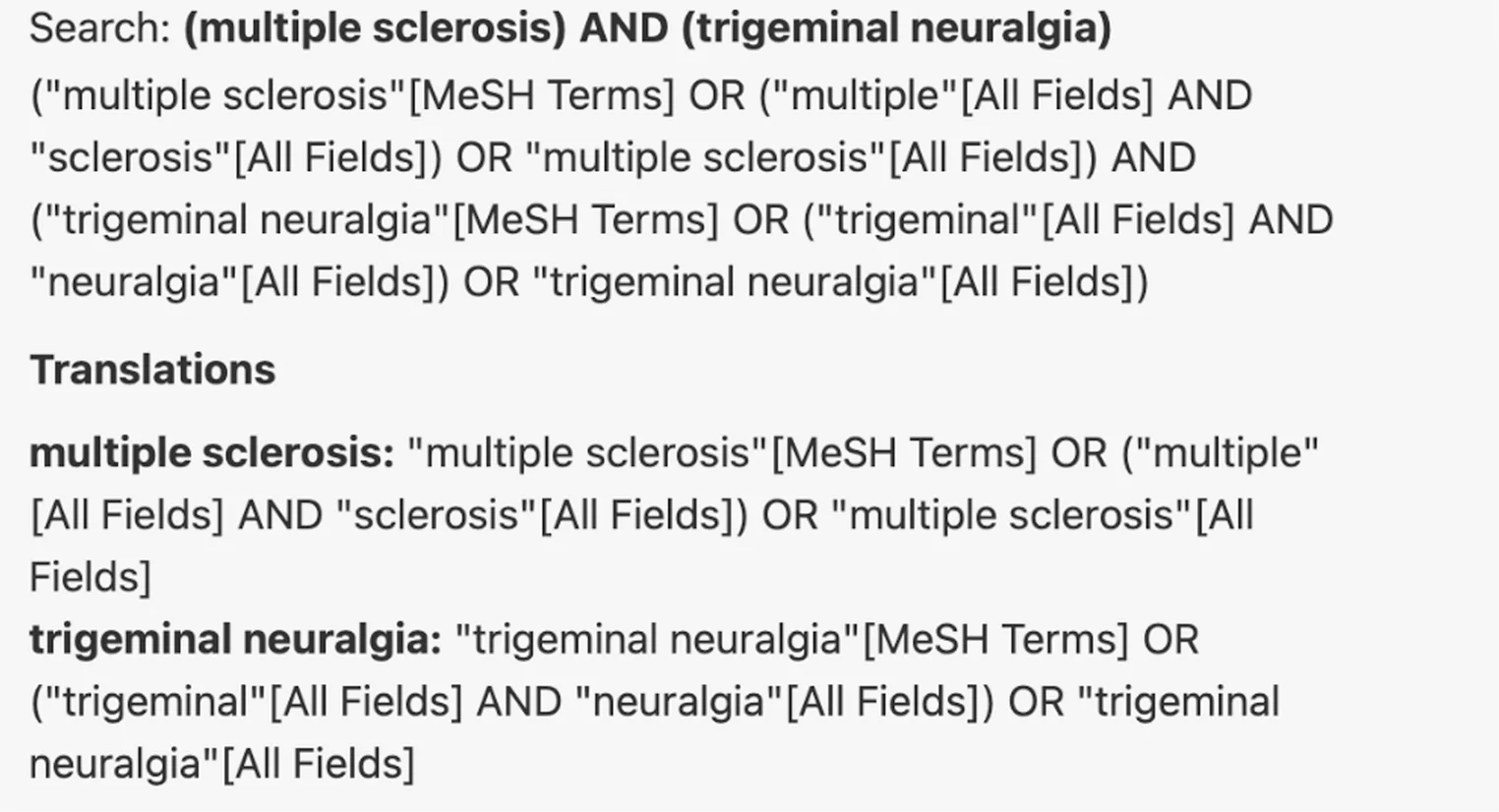
Search Terms used for database searches in PubMed, Embase, and Scopus with a representation of how PubMed specifically processed the search terms. MeSH = Medical Subject Headings.

Studies were sourced from PubMed, Scopus, and Embase, chosen for their broad biomedical coverage, reproducibility, and strength in evidence synthesis [29–31]. The search terms used for these databases are shown in Fig. 1. No registers were used, and database searches were conducted between October 1, 2024, and April 14, 2026.

### Study eligibility criteria

Studies were included if they met *all* of the following criteria:

- The study analysed the efficacy of at least one surgical treatment modality for trigeminal neuralgia.
- The study featured a defined MS-TN cohort where the analysed patient cohort had trigeminal neuralgia and a clinically confirmed diagnosis of MS.
- The study investigated the pain relief of each intervention using the BNI pain scale or a variant of this.
- The study published post-intervention pain relief outcomes, defined and distinguished for the MS-TN cohort.
- The study was either a case-control study, cohort study or randomised control trial.
- The study was published in English.

Studies were excluded if they consisted of *any* of the following:

- Case Reports or Case Series.
- Systematic and Literature Reviews.
- Studies published prior to 1st January 2000.
- Opinion Pieces or Editorials.
- Non-Human Studies.
- Studies with paediatric subjects (under the age of 18).

### Selection process

Studies published before 2000 were excluded as high-resolution MRI sequences capable of reliably identifying NVC (e.g., FIESTA) became widely clinically available around 2000 [32, 33] which is directly relevant to patient selection in MS-TN where vascular conflict and demyelinating pathophysiology may co-exist. Furthermore, the introduction of the McDonald Criteria in 2001 marked a significant step toward standardised MS diagnosis, reducing the diagnostic heterogeneity seen in earlier literature [34]. Including earlier studies would have introduced methodological heterogeneity without meaningfully improving applicability to contemporary clinical practice. Case reports were excluded due to the high risk of bias [35] whilst excluding secondary reviews ensured analysis of first-hand data.

Embase, PubMed and Scopus were searched by AK. After duplicate removal using EndNote and manual verification, titles and abstracts were independently screened against the inclusion and exclusion criteria by three reviewers (AK, DC, SK) using Rayyan. Full-text review of potentially eligible studies was then performed independently by the same reviewers, with any disagreements at either stage resolved by consensus.

### Barrow neurological institute pain intensity score

The Barrow Neurological Institute (BNI) pain score consists of five categories (I-V) as shown in Table 1 with post-interventional scores of I-III indicating success and scores of indicating IV-V failure [37]. It notably standardizes outcomes across interventions and accounts for post-surgical medication changes [38–40].

**Table 1:** Barrow Neurological Institute Pain Intensity Score

BNI Score	Pain Evaluation
I	No pain, no medication
II	Occasional pain, not requiring medication
III	Some pain, adequately controlled with medication
IV	Some pain, not adequately controlled with medication
V	Severe pain/no pain relief

BNI Pain Scale - adapted from Reddy et al. [36]. BNI = Barrow Neurological Institute

### Data collection / outcomes

Data was extracted to address predefined primary and secondary research questions. The primary outcome was the efficacy of surgical interventions (MVD, PBC, RFL, GKR, INL, PSR) in achieving initial and sustained pain relief (BNI I-III) in patients with MS-TN.

Secondary outcomes included postoperative adverse events, associations between MS subtype and pain outcomes, and the relationship between GKR radiation dose and successful pain control (BNI I-III).

Extracted variables included patient demographics, study design, intervention characteristics (including GKR dosages), pain outcomes at initial and final follow-up, and complications.

### Risk of bias assessment

The methodological quality of included cohort and case-control studies was assessed using the Newcastle-Ottawa Scale (NOS) [41]. Three reviewers (AK, DC, SK) independently appraised each study, with discrepancies resolved by consensus following calibration exercises on sample studies. Studies were classified as high (7–9 stars), moderate (4–6 stars), or poor quality (0–3 stars).

This systematic review originated as an undergraduate degree (intercalated BSc) dissertation by author AK. As no other authors were permitted as part of that dissertation, prospective registration in PROSPERO as a systematic review was not possible, and subsequent retrospective registration is not recommended. Therefore, this systematic review has not been registered.

As a result of clinical heterogeneity in the identified studies and the relatively small and uneven number of studies for each modality, neither meta-analysis or sensitivity analysis was performed.

## Results

**Fig. 2:**
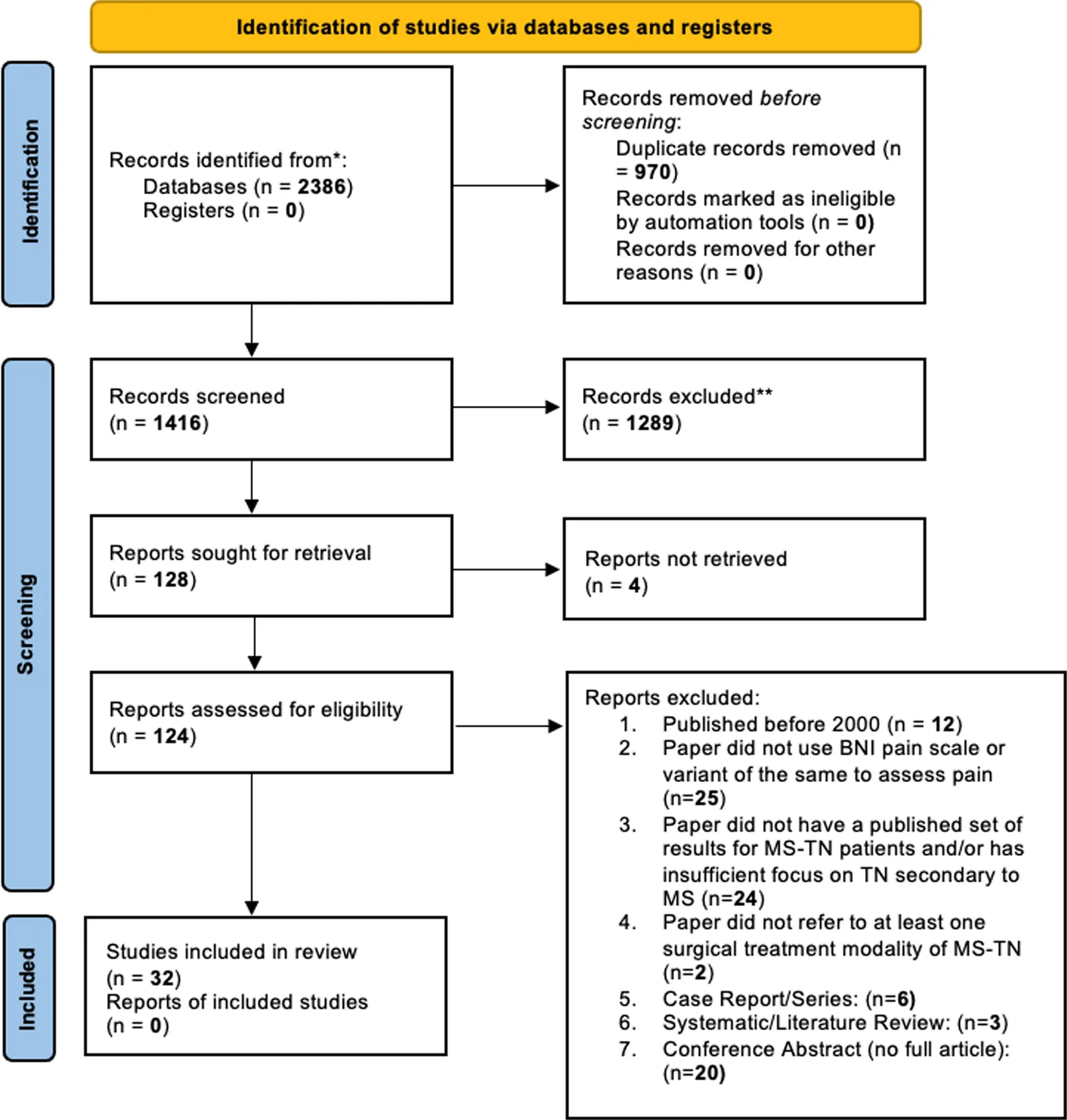
PRISMA flow diagram describing the sequential identification and screening process of articles in this study (Page et al. [42]). BNI = Barrow Neurological Institute; MS-TN = Trigeminal Neuralgia secondary to Multiple Sclerosis; TN = Trigeminal Neuralgia; MS = Multiple Sclerosis *Refers to sum of records across all databases (Scopus, Embase and PubMed); ** All reports were excluded by the authors and double-checked with automated tools (EndNote)

**Fig. 3:**
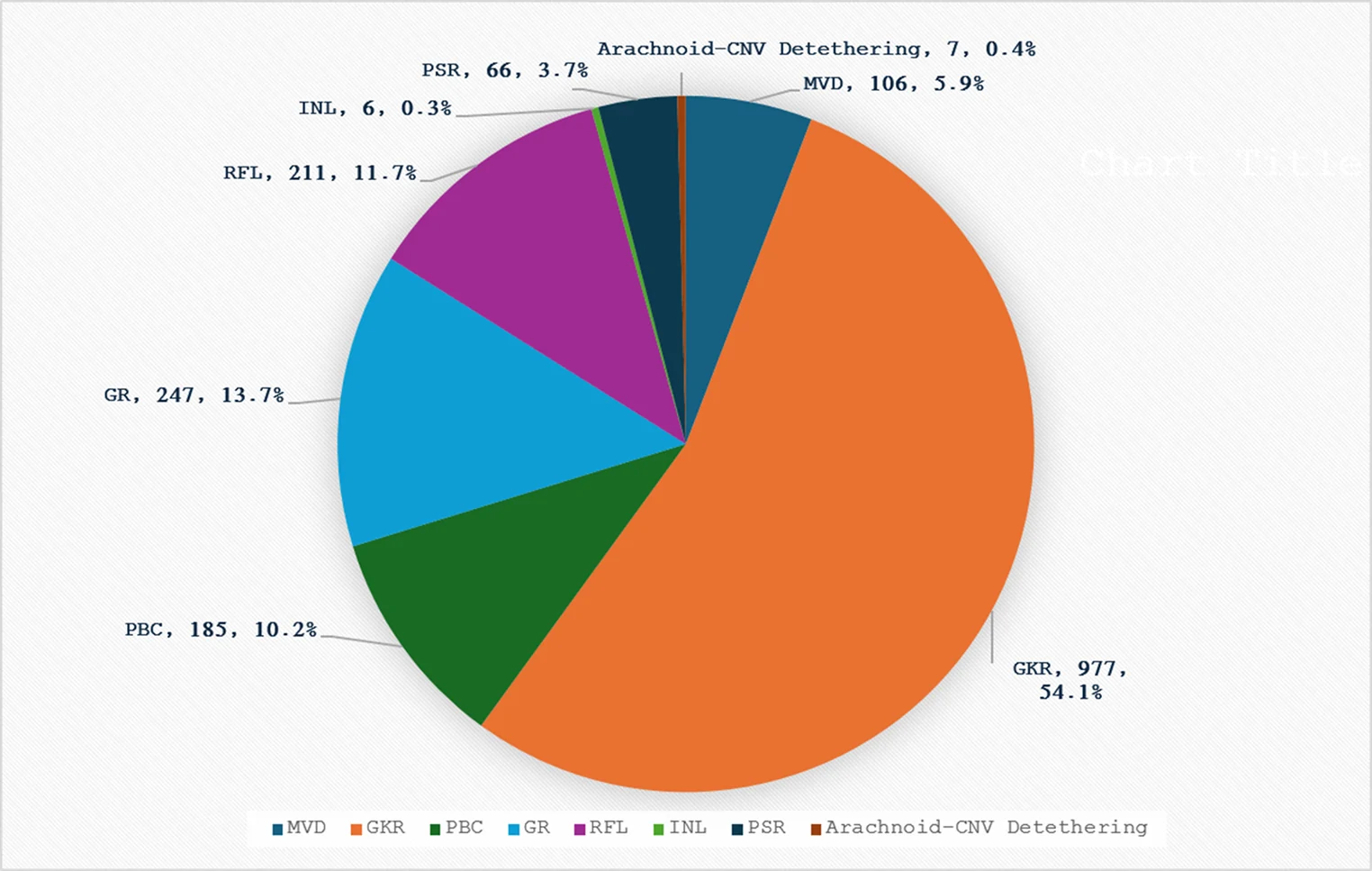
Pie chart showing the total number of patients receiving each treatment in the included studies. The percentage of patients for each treatment is given in brackets relative to the total number of patients across all studies. MVD = Microvascular Decompression; GKR = Gamma Knife Radiosurgery; PBC = Percutaneous Balloon Compression; GR = Glycerol Rhizotomy; RFL = Radiofrequency Lesioning; INL = Internal Neurolysis; PSR = Partial Sensory Rhizotomy

Database searches yielded 605 (PubMed), 858 (Scopus), and 923 (Embase) studies, totalling 2,386 studies overall. After removing 970 duplicates, 1,416 records were screened by title and abstract, identifying 128 potentially relevant studies; four could not be retrieved, leaving 124 for full-text review. Applying the inclusion and exclusion criteria resulted in 32 studies included, as illustrated in the PRISMA-type flowchart (Fig. 2). The 32 studies encompassed a total of 1617 patients and 1805 procedures across eight surgical interventions, as highlighted in Fig. 3.

GKR accounted for 54.1% of all procedures, whilst INL made up only 0.3%. GKR featured in 17 studies, RFL in seven studies, PBC and MVD in six studies each, GR in five studies, INL and PSR in three studies each and arachnoid-CNV detethering in one study. Table 2 summarises patient demographics, with studies conducted across 13 high-income countries in North America, Europe, and Asia Table 2.

**Table 2:** Table showing the demographics and study details of each article

Author and Year	Country of Origin of Study	Patient Demographics and Study Design	Temporal Relationships between TN, MS and Intervention
Zorro et al., [53]	USA	Mean Age of MS-TN Patients: 59 (range: 38–74) Gender of MS-TN Patients (M/F): (10/27) Median Follow Up Period (months): 56.7 (range: 6-174)	Mean TN Duration prior to GKR in MS-TN (years): 5 (range: 2–20) Mean duration of MS prior to GKR in MS-TN (years): 15 (range: 2–46)
Verheul et al., [69]	Netherlands	Mean Age of All Patients: 65 (range: 27–91) Gender of All Patients (M/F): (164/201) Median Follow Up Period (months): 28	
Montano et al., [67]	Italy	Mean Age of MS-TN Patients: 52.23 ± 11.26 Gender of MS-TN Patients (M/F): (10/11) Mean Follow Up Period (months): 51.57 ± 20.89	Mean MS Duration prior to procedure (years): 13.81 ± 8.41 Mean TN Duration prior to procedure (years): 5.61 ± 4.51
Mathieu et al., [61]	Canada	Mean Age of MS-TN Patients (GKR): 59 (range: 42–78) Mean Age of MS-TN Patients (GR): 56.5 (range: 41–79) Gender of MS-TN Patients (M/F): (21/24) Median Follow Up Period (months): 39 (range: 13–69)	Mean TN Duration prior to GKR in MS-TN (months): 60 (range: 12–276) Mean TN Duration prior to GR in MS-TN (months): 38 (range: 2–75)
Tuleasca et al., [66]	France	Median Age of MS-TN Patients: 57.2 (range: 36.1–82.6) Gender of MS-TN Patients (M/F): (23/20) Median Follow Up Period (months): 53.8 (range: 12-157.1)	Median TN Symptom Duration to Time of Surgery for All Patients (months): 72 months (range: 2-390)
Weller et al., [59]	USA	Median Age of MS-TN Patients: 62 (range: 39–86) Gender of MS-TN Patients (M/F): (14/21) Median Follow Up Period (months): 39 (range: 3–97)	Median Duration from Diagnosis to Treatment (years): 3 (range: 0–25)
Krishnan et al., [63]	Canada	Mean Age of MS-TN Patients undergoing GKR: 56 Mean Age of MS-TN Patients undergoing GR/PBC: 57 Gender of MS-TN Patients (M/F): (8/13) Median Follow Up Period (months): 59 (range: 13–158)	Mean age at TN diagnosis (years): 53 ± 8
Rashid et al., [68]	Germany	Mean Age of All Patients: 66 (range: 36–93) -Mean Age of Classical TN Patients: 73 -Mean Age of MS-TN Patients: 55 Gender of All Patients (M/F): (27/28) -Gender of MS-TN Patients (M/F): 6/9 Mean Follow Up Period for All Patients (months): 30	
Przybylowski et al., 2018 [60]	USA	Mean Age of MS-TN Patients: 42 (range: 18–66) Gender of MS-TN Patients (M/F): (11/31) Mean Follow Up Period (months): 78 (range: 36–226)	
Asplund et al., [62]	Sweden	Mean Age of MS-TN Patients: 61 (range: 42–82) Gender of MS-TN Patients (M/F)(%): 31/69 Mean Follow Up Period (months): 19 (range: 0-122)	Mean pre-operative duration of MS (years): 24.7 Mean pre-operative duration of TN (years): 10.6
Xu et al., 2019 [58]	USA, Canada, China	Median Age of MS-TN Patients: 57 (range: 31–90) Gender of MS-TN Patients (M/F): (81/182) Median Follow Up Period (months): 17 (range: 0.5–144)	
Noorani et al., [65]	UK	Median Age of MS-TN Patients: -GR: 61.1 -PBC: 59 -RFA: 62.3 Gender of MS-TN Patients (M/F): -GR: 11/8 -PBC: 4/12 -RFA: 8/21 Mean Follow Up Period (months): 24.6	Mean Duration of TN symptoms in MS-TN patients (years): 8.6
Staudt et al., [24]	Canada	Mean Age of ITN Patients at first GR: 69.9 ± 12.8 Mean Age of ITN Patients at repeat GR: 74 ± 12.1 Mean Age of MS-TN Patients at first GR: 55.4 ± 8.8 Mean Age of MS-TN Patients at repeat GR: 57.9 ± 8.5 Gender of MS-TN Patients at first GR (M/F): (27/51) Gender of MS-TN Patients at repeat GR (M/F): (39/65) Mean Follow Up Period for All Patients (years): 2.5 ± 3.7	Mean MS Duration prior to first GR (years): 15.6 ± 9.4 Mean MS Duration prior to repeat GR (years): 19.5 ± 9.6 Mean TN Duration prior to first GR in MS-TN (years): 4.2 ± 3.8 Mean TN Duration prior to repeat GR in MS-TN (years): 2 ± 2.7
Bigder et al., [45]	Canada	Mean Age of MS-TN Patients: 59.5 (range: 48–76) Gender of MS-TN Patients (M/F): (3/9) Mean Follow Up Period (months): 36.5 (range 1-130)	Mean TN Duration prior to MSR in MS-TN (years): 7.8 (range: 0.8–18.2)
Paulo et al., [16]	USA	Mean Age of MS-TN Patients: 53.1 ± 2.1 Gender of MS-TN Patients (M/F): (6/27) Mean Follow Up Period (months): 53.5	Mean MS Duration prior to MVD in MS-TN (years): 11.8 ± 1.6 Mean TN Duration prior to MVD in MS-TN (years): 6.6 ± 1.04
Lee et al., [64]	USA	Mean Age of MS-TN Patients (GKR): 61 (range: 39–96) Mean Age of MS-TN Patients (RFA): 57 (range: 35–77) Gender of MS-TN Patients (M/F): 10/32 Median Follow Up Period (months): 110 (GKR)/16 (RFA)	Mean Duration of Pain prior to Treatment (months) GKR: 105 (range: 1-432) Mean Duration of Pain prior to Treatment (months) RFA: 62 (range: 9-156)
Mousavi et al., [47]	USA	Median Age of MS-TN Patients: 60 (range: 30–79) Gender of MS-TN Patients (M/F): (22/29) Follow Up Period: 1, 3 and 6 years	Median MS Duration until TN diagnosis (years): 14 (range: 1–45) Median Duration of TN until RFL (years): 4 (range: 1–30)
Guillemette et al., [52]	Canada	Median Age of All Patients: 68.5 (range 23–90) Gender of All Patients (M/F): (60/108) Median Follow Up Period (months): 36.7 (range: 0.8-192.2)	
Durnford et al., [55]	UK	Median Age of All Patients: 55 (range: 31–77) Gender of All Patients (M/F): (1/7) Median Follow Up Period (months): 38 (range: 24–43)	Median TN Symptom Duration to Time of Surgery for All Patients (months): 69 months (range: 14–219)
Worm et al., [43]	Denmark	Mean Age of MS-TN Patients: 54.61 Mean Age at TN Onset: 46.33 Gender of MS-TN Patients (M/F): (7/11) Follow Up Period: 3, 6 and 12 months	Mean MS Duration prior to intervention (years): 15.89 (range 0–29) Mean TN Duration prior to intervention (years): 7.7 (range: 0–27)
Gündüz et al., [48]	Turkey	Mean Age of All Patients: 57.66 (range: 27–89; SD: ±14.05) Gender of All Patients (M/F): (92/123) Mean Age of MS-TN Patients: 49.46 (range: 34–67; SD: ±11.01) Gender of MS-TN Patients (M/F): (8/5) Mean Age of MS Onset: 37.5 (SD: ±8.07) Follow Up Period (months): 36	Mean Interval between MS Diagnosis and onset of TN symptoms (years): 8.8 (SD: ±4.71)
Leduc et al., [50]	Canada	Median Age of MS-TN Patients: 57 (range: 34–79) Gender of MS-TN Patients (M/F): (27/50) Median Age of Control (Idiopathic/Classical TN) Patients: 59 (range: 32–91) Gender of Control Patients (M/F): (26/51) Median Follow Up Period (months): 23	
Kalluri et al., [56]	USA	Mean Age of MS-TN Patients: -55 (SD ± 10.2) overall -54 (SD ± 10.0) RRMS -58.3 (SD ± 9.2) SPMS and PPMS Gender of MS-TN Patients (M/F): -(16/29) overall -(10/24) RRMS -(6/5) SPMS and PPMS Median Follow Up Period (months): 30.1	Mean duration of MS/TN prior to MVD (months): -197.6/86.4 (SD ± 132.6/±84.9) overall -184.6/89.6 (SD ± 116.6/±92.6) RRMS -237.6/76.5 (SD ± 180.8/±73.3) PPMS and SPMS
Öztürk Özlük et al., [54]	Turkey	Median / Mean Age of MS-TN Patients: 52 (range: 39–76) / 52.57 (SD: ±10.67) Gender of MS-TN Patients (M/F): (10/18) Median / Mean Follow Up Period (months): 27 (range: 12–181) / 44.18 (SD: ±44.3)	Median Disease Duration of TN (months): 60 (range: 18–240) Median Disease Duration of MS (months): 96 (range: 48–360)
Lozouet et al., [49]	France	Mean Age of non-MS-TN patients: 64.4 (SD: ±13.9) (Range: 31–89) Mean Age of MS-TN Patients: 56.6 (SD: ±9.9) (Range: 36–72) Gender of MS-TN Patients (M/F): (6/13) Mean Follow Up Period (months): 48	
Franzini et al., [51]	Italy	Median Age of MS-TN Patients: 58.5 (IQR: 53–65) Median Age at Initial GKR: 54.5 (IQR: 49–61) Gender of MS-TN Patients (M/F): (6/12) Median Follow Up Period (months): 36 (IQR: 22–56)	Median duration of MS before repeat GKR (years): 23 (IQR: 14–28) Time Between initial and repeat GKR (months): 22.5 (IQR: 12–46)
Mazzapicchi et al., [71]	Italy	Mean Age of MS-TN Patients: 64 (SD: ±14) (range: 34–96) Gender of MS-TN Patients (M/F): (6/29) Mean Age at MS Diagnosis: 39 (SD: ±13.4) (range: 15–72) Mean Age at Operation: 44.6 (SD: ±16) Follow Up Period (months): 77 (SD: ±68.7)	Mean Duration of Pain prior to Treatment (months): 61.1 (SD: ±60) (range: 5-240)
De Nigris Vasconcellos et al., [57]	USA, Canada, Spain, Italy, Turkey, Czech Republic	Median Age of MS-TN Patients: 57 (range: 32–91) Gender of MS-TN Patients (M/F): -(78/180) Overall -(40/95) RRMS -(10/20) PPMS -(28/65) SPMS Median Follow Up Period (months): 36	Median Time since MS/TN Diagnosis (years): -15/3 RRMS -11/4 PPMS -18/4 SPMS
Sozer et al., [70]	Turkey	Mean Age of MS-TN Patients: 55.41 ± 9.86 Gender of MS-TN Patients (M/F): (13/24) Median Follow Up Period (months): 81.19 ± 44.40	
Bassiouni, Botsch and Zouba, [17]	Germany	Mean Age of All Patients: 56.7 ± 14.6 Gender of All Cases (M/F): (13/24) Median Follow Up Period (months): 24 (range: 7–46)	
Joncas et al., [46]	USA	Mean Age of MS-TN Patients: 59.6 ± 9.1 Gender of MS-TN Cases (M/F): (6/31) Median Follow Up Period (years): 3.14 ± 2.58	Mean Preoperative TN Duration MS-TN (years): 7.33 ± 4.81
Lange et al., [44]	Germany	Mean Age of All Patients: 59.4 Gender of MS-TN Cases (M/F): (9/6) Gender of All Cases (M/F): (22/26) Mean Follow Up Period (months): 38 (range: 3-183)	Mean Pre-operative TN Duration in All Patients (years): 7.81 (range 1–30)

Legend: *MS-TN* Trigeminal neuralgia secondary to multiple sclerosis, *TN* Trigeminal neuralgia, *MS* Multiple sclerosis, *RFL* Radiofrequency lesioning, *GKR* Gamma knife radiosurgery, *M/F* Male/Female, *RRMS* Relapsing remitting multiple sclerosis, *PPMS* Primary progressive multiple sclerosis, *SPMS* Secondary progressive multiple sclerosis, *SD* Standard deviation, *IQR* Interquartile range

### Pain outcomes

Across the included studies, all surgical modalities provided meaningful initial pain relief in MS-TN, but durability and complication profiles varied considerably.

The full breakdown of the key findings from each article is reported in Table 3. The study by Worm et al. [43] reported satisfactory outcomes after PBC/GR in 64% of MS patients and after MVD in 71%, but MVD carried more frequent and more severe complications than percutaneous options. PSR demonstrated excellent efficacy and durability in the study by Lange et al. [44] noting significant improvement in BNI scores at final follow-up (*p* < 0.0001). Bigder et al. [45] specifically highlighted the superiority of PSR compared to percutaneous procedures and GKR. PSR was also described with improved pain outcomes when utilised in multimodality treatment regimens alongside MVD and INL [46]. In the single study identified in this review examining arachnoid-CNV conflict, targeted release proved effective with 75% achieving BNI I at 24 months. The one patient who required a repeat procedure (performed for persistent arachnoid strands), achieved BNI I post-revision Table 3.

**Table 3:** Table highlighting the interventions analysed and the key findings of each study

Author and Year	Primary Intervention(s) Studied	Study Design	Number of MS Patients / Total Number	Summary of Main Findings	Recommendations and Conclusions
Zorro et al., [53]	GKR	Retrospective Cohort	37/37	-97.2% and 62.1% achieved BNI I-III at initial and final follow-up respectively. 37.8% experienced recurrence at a median time of 74.5 months. -Age > 60 was associated with higher recurrence (*p* = 0.046); prior surgical management (*p* = 0.673), MRI findings and maximum dose was not associated with pain recurrence. -Only 5.4% developed some degree of facial numbness.	-GKR has a very low morbidity and represents a satisfactory treatment strategy for MS-TN.
Verheul et al., [69]	GKR	Retrospective Cohort	31/365	-Probability of achieving long-term pain relief was significantly worse in MS-TN patients than with ITN (*p* < 0.01); more treatments to achieve adequate pain relief required in MS-TN cohort. -Earlier pain recurrence in MS-TN did not reach statistical significance (*p* = 0.1) -No statistical difference in post-operative numbness between MS-TN and ITN cohorts.	-Long term treatment outcomes are worse in MS-TN compared to ITN; repeat GKR, due to its non-invasive nature and limited additive morbidity, make GKR an effective treatment option in MS-TN.
Montano et al., [67]	PBC	Retrospective Cohort	21/21	-80.95% reported acute pain relief; 57.14% experienced recurrence with a mean pain-free survival rate of 15 months. -Repeat PBC allowed for maintenance of adequate pain control (BNI I-III). -The presence of a single affected CNV division (*p* = 0.042), no prior operations (*p* = 0.048), compression time < 5 min (*p* = 0.0067) and pear-shaped balloon (*p* < 0.05) were predictors of higher pain-free survival.	-PBC’s low complication profile combined with its easily repeatable nature support its role as first line in drug-resistant MS-TN. -Operative technique should be examined further to optimise outcomes.
Mathieu et al., [61]	GKR, GR	Retrospective Cohort	45/45	-GR offered superior initial and final pain outcomes compared to GKR with lower recurrence rates (despite earlier median time to recurrence). -GKR was safer than GR with lower rates of sensory side effects. -Higher treatment doses (> 80gy) were associated with increased likelihood of pain relief and lower rates of recurrence (*p* = 0.003; 0.002 respectively).	-GKR and GR both provide satisfactory treatment strategies for refractory MS-TN. -GR should be favoured in MS-TN patients in acute pain with treatment choice in other patients depending on patient preference and expectations.
Tuleasca et al., [66]	GKR	Prospective Cohort	43/43	-90.7% had initial pain cessation (BNI I) in a median time of 30 days. -61.5% experienced pain recurrence; median time to recurrence of 16 months. -Age at GKR, laterality and location of pain, time elapsed until treatment, prior surgery and side effects were not statistically predictive of pain outcomes (*p* = 0.09–0.49). -Presence of NVC was a positive predictor of better pain outcomes (*p* = 0.03).	-GKR represents a safe and effective treatment modality for MS-TN with a low rate of numbness (never bothersome or debilitating) despite high recurrence rates.
Weller et al., [59]	GKR	Retrospective Cohort	35/35	-82% initial response rate to GKR (BNI I-III) with a median time to pain relief of 42 days. -40.7% experienced pain recurrence at a median interval of 9.6 months. -Gender and age did not predict treatment efficacy; facial numbness was also not predictive (*p* = 0.62). -GKR dose and nerve length were not predictive of post operative numbness.	-GKR represents a safe alternative in MS-TN management with no bothersome facial sensory disturbance noted.
Krishnan et al., [63]	GKR, PBC, GR	Retrospective Cohort	21/21	-Initial pain relief was experienced by 92% of GKR patients (12/13) and 77% of PBC/GR patients (10/13) (*p* = 0.3). -However, 88% of all treatments (85% GKR/92% PBC/GR) failed at final follow up (BNI IV-V); the average time to fail did not statistically differ between treatment modality (*p* = 0.64). 73 subsequent surgeries were required for the remaining 23 patients.	-A high failure rate for standard rhizotomies was noted with multiple retreatments necessary.
Rashid et al., [68]	GKR	Retrospective Cohort	15/55	-In MS-TN compared to classical TN, the BNI I-III rate was significantly lower at both initial and final follow-up (*p* = 0.03;0.003 respectively). -The rate of new hypaesthesia post-GKR was not statistically different between classical and MS-TN cohorts.	-Whilst notably not a curative option, GKR remains a valid treatment strategy due to its acceptable efficacy in MS-TN and pretreated cases.
Przybylowski et al., [60]	GKR	Retrospective Cohort	42/42	-With index GKR alone, only 7% of patients obtained BNI I with 26.2% achieving BNI I-III. -Following multimodal salvage therapies (occurring at a median of 13 months after initial therapy), 35/42 (83%) were able to achieve pain control. -Treatment dose was not a statistically significant predictor of pain outcomes.	-GKR offers limited but acceptable short-term pain control for MS-TN, but multimodality treatment is required for long-term pain relief. -Salvage GKR can offer beneficial pain control with low rates of bothersome facial numbness.
Asplund et al., [62]	PBC	Retrospective Cohort	66/66	-Inconsistent efficacy across individual – some cases experience durable pain relief with other case demanding several less satisfactory subsequent treatments. -Sex, age, MS duration, TN duration, pain side, maximum pain branch and prior surgery did not significantly predict time to recurrence (*p* = 0.15–0.97). -Overall decrease in therapeutic effect with an increasing number of prior PBCs – e.g., superior pain relief maintenance following first treatment compared to fourth treatment (*p* = 0.009) and fifth treatment (*p* = 0.034).	-PBC represents a minimally invasive and safe treatment strategy for refractory MS-TN. -Reconsideration of management strategy following one or two-failed PBCs requires investigation.
Xu et al., [58]	GKR	Retrospective Cohort	263/263	-88.2% achieved pain control with a median maintenance time interval of 14.1 months. -Statistically significant correlation between achieving BNI I at initial follow up and maintenance of a pain-free interval (*p* = 0.046). -No correlation noted between age at initial GKR, gender, laterality or maximum dosage and initial and final pain outcomes (*p* = 0.14–0.94; *p* = 0.27-3 respectively). -No statistically significant difference regarding maintenance of pain control in those treated with an upfront or secondary GKR (*p* = 0.487).	-SRS was a recommended as a first-line or adjunct for MS-TN given its favourable low-risk profile.
Noorani et al., [65]	PBC, RFA, GR	Retrospective Cohort	33/204	-No significant difference in overall initial pain outcomes between the modalities despite a higher rate of BNI V post-GR (GR: 21.1%; RFA (3.4%); PBC (6.3%)). -53.1% and 59.3% initial BNI I-III in MS-TN and ITN cohorts respectively with a shorter median time to pain recurrence in MS-TN. -Similar complication rates between the three modalities in the MS-TN cohort.	-PBC and RFA delivered superior results compared to GR with regards to relief durability. The safety and efficacy of PBC and RFA support their use as first-line surgical options in MS-TN.
Staudt et al., [24]	GR	Retrospective Cohort	182/401	-No statistical difference in initial pain outcomes or in median time to pain recurrence between primary GR in MS-TN and ITN cohorts (*p* = 0.726; 0.87 respectively). -With repeat GR, statistically significant prolonged pain-free intervals were observed in MS-TN compared to repeat GR in ITN (*p* < 0.05). -Presence of NVC had no impact on pain outcomes; subsequent hypoesthesia was highly prognostic of pain free survival (*p* < 0.01).	-MS-TN cohorts can achieve meaningful and lasting pain relief following GR with efficacy and durability comparable to ITN. -Initial and subsequent GR procedures in MS-TN are equally efficacious and represent an effective treatment strategy.
Bigder et al., [45]	PSR	Retrospective Cohort	12/30	-85.7% of PSR cohort achieved BNI I at initial follow-up; 77% of MSR patients remained at BNI I at final follow-up compared to 29% of non-MSR cohort (*p* < 0.001). -PSR consistently provided a longer time to treatment failure compared to percutaneous procedures and GKR (79 vs. 10 months; *p* = 0.0007). -No major operative complications or adverse neurological changes noted post-PSR.	-PSR offers a highly effective and durable option for MS-TN cohorts, with superior outcomes to percutaneous procedures and GKR. -PSR should be considered earlier in the disease course for refractory MS-TN.
Paulo et al., [16]	MVD	Retrospective Cohort	33/33	-67% reported some degree of pain relief following MVD (40% BNI I). -Sex, MS subtype, presence of MS plaques or vessel compression did not predict post-operative pain outcomes. -Higher pre-operative BNI score was associated with unfavourable outcomes (*p* = 0.006).	-With no radiographic evidence of plaque formation near CNV, MVD may deliver beneficial outcomes to this specific subset of MS-TN patients.
Lee et al., [64]	GKR, RFA	Retrospective Cohort	42/42	-At final follow-up, although overall BNI outcomes were not statistically different (*p* = 0.154), RFA achieved higher BNI I rate compared to SRS (*p* = 0.031). -In those who achieved BNI I-III, RFA resulted in faster median onset of pain freedom (*p* < 0.001) whereas GKR patients were significantly more likely to be pain-free at 2 years (*p* = 0.044). -There were no differences in paraesthesia or dysesthesia between the GKR or RFA treatment groups (*p* = 0.067;*p* = 0.890 respectively). -RFA patients with paraesthesia had a significantly shorter time to pain recurrence (*p* < 0.01). -Procedures per patient were similar between GKR and RFA (1.17 vs. 1.31; *p* = 0.012).	-Both GKR and RFA are valid treatment options in MS-TN with similar initial and final pain outcomes. -SRS affords longer duration of pain-freedom whereas RFA provides more immediate pain-freedom.
Mousavi et al., [47]	RFL	Single-centre retrospective cohort study	51/51	-MS-TN was highly responsive to RFL, but pain recurrence was common with repeat procedures necessary for maintenance – 23/51 patients underwent repeat RFL. -No statistically significant difference in pain relief outcome noted between patients who had prior procedures vs. those with primary RFL (*p* = 0.97).	-RFL was deemed a viable outpatient procedure with brief sedation and minimal complication rates. -Repeat RFL had similar efficacy when compared to initial RFL.
Guillemette et al., [52]	GKR	Single-centre retrospective cohort study	44/166	-Older age (*p* < 0.001) and shorter symptom duration prior to treatment (*p* = 0.006) were the only positive treatment outcome predictors. -Patients with an onset or aggravation of hypoesthesia post-intervention were more likely to achieve longer adequate pain control (*p* < 0.001). -Pain relief was maintained for longer periods of time in primary vs. MS-TN (*p* < 0.001).	-GKR was favourable for medically refractory MS-TN cases with a low risk of incapacitating complications.
Durnford et al., [55]	INL	Single-centre retrospective cohort study	2/8	-INL did not target any pontine demyelination with no pain recurrence in MS-TN.	-INL proved to be a safe and highly effective short-term treatment for refractory MS-TN; longer term efficacy remains unknown.
Worm et al., [43]	MVD, PBC, GR	Single-centre prospective cohort study	18/18	-MVD in MS-TN had higher complication rates and lower efficacy than in primary TN. -Outcomes and complication rates of percutaneous procedures in the MS-TN cohort were deemed comparable to MVD in classical and idiopathic TN. -64% and 71% of patients had satisfactory outcomes post-PBC/GR and post-MVD respectively but MVD featured more severe and frequent complications.	-Percutaneous procedures were recommended as first-choice surgical interventions for MS-TN. -MVD was only recommended when NVC with morphological changes was present.
Gündüz et al., [48]	RFL	Single-centre retrospective cohort study	13/215	-Mean remission time and pain-free period of MS-TN patients were significantly shorter than in primary TN, requiring more subsequent interventions (*p* < 0.0001). -No statistically significant difference (*p* = 0.90) noted between the idiopathic TN and MS-TN cohorts in terms of satisfactory early treatment outcomes (93.1% vs. 96.43% respectively) and complications.	-RFL of Gasserian ganglion was effective, reproducible and had few complications in the typical primary TN cohort with longer-acting treatment modalities notably demanding further investigation in the MS-TN cohort.
Leduc et al., [50]	GKR	Single-centre retrospective case-control study	77/144	-No significant difference noted in initial pain relief rates post-GKR between MS-TN patients and primary TN controls respectively (77% vs. 69%; *p* = 0.889) or in the median time to initial pain freedom (17 weeks vs. 12 weeks; *p* = 0.385). -MS-TN was associated with significantly earlier pain recurrence (29 months) compared to primary TN (75 months) (*p* = 0.002). -78% of MS cases saw recurrence compared to 52% of controls. -mBNI I was seen in 34% of MS-TN patients vs. 49% for controls (*p* = 0.007).	-GKR is safe and effective for refractory MS-TN featuring similar initial pain-relief rates compared to classical TN despite earlier recurrence/shorter pain-free intervals. -GKR timing in MS-TN patients should be carefully planned to allow for maximal benefit without compromising feasibility of future interventions.
Kalluri et al., [56]	MVD	Single-centre retrospective cohort study	45/45	-In both the RRMS cohort and the combined primary/secondary progressive cohort, orofacial pain was significantly reduced at all time points following MVD (*p* < 0.001). -Only disease-modifying therapy use affected final follow-up BNI pain score; use was significantly associated with numbness at final follow-up (*p* = 0.01). -Shorter post-operative pain free interval found in RRMS patients (7.8 months compared to 55.6 months for progressive MS-TN patients; *p* = 0.04).	-No significant relationship noted between MS subtype and final follow-up BNI score. -RRMS patients were significantly more likely to suffer pain recurrence. (*p* = 0.02) and worse outcomes. -The degree of post-operative pain relief between RRMS and progressive MS patients did not differ.
Öztürk Özlük et al., [54]	GKR	Single-centre retrospective cohort study	28/28	-GKR harboured similar complications to other ablative therapies in MS-TN patients; trigeminal nerve sensory dysfunction was the most common complication, notably reducing over time.	-GKR was an effective treatment modality in MS-TN patients not expected to achieve acute pain relief from other treatment modalities (medications, other ablative procedures and surgery); GKR was thus suggested in patients with insufficient pain relief following other interventions.
Lozouet et al., [49]	PBC, RFL	Single-centre retrospective cohort study	19/72	-Pain recurrence was more common and frequent in MS-TN than non-MS patients (*p* < 0.05) with more procedures per patient in the MS-TN cohort (*p* = 0.0036) -No significant differences in favourable BNI scores at final follow-up between MS and non-MS groups (*p* = 0.76). -Similar complication rates for both MS-TN and primary TN patients.	-RFL and PBC showed satisfactory pain relief in MS-TN patients despite higher recurrence rates and necessity for subsequent procedures.
Franzini et al., [51]	(Repeat) GKR	Single-centre retrospective cohort study	18/18	-Repeat GKR achieved duration of pain relief in MS-TN patients within a median period of 21 days (IQR 14–60 days) as seen in 15/18 patients. -50% were estimated to have pain reduction at a 7 year follow-up on a Kaplan-Meier survival curve. -Pain recurred in 5/15 patients after a median period of 12 months (IQR: 3–12 months). -For all patients, sex, MS duration, maximum radiation dose and the presence of MRI-evident demyelination plaques did not predict pain reduction.	-Repeat GKR is an effective strategy in prolonging duration of pain relief. -Older age was a predictor of sustained pain reduction after repeat GKR (*p* = 0.01) and could be a factor to consider in patient selection for a second procedure (older age at primary GKR did not predict a longer period of pain relief/reduction).
Mazzapicchi et al., [71]	MVD, INL, PBC, GKR, RFL	Single-centre retrospective cohort study	35/35	-MVD and/or INL were used for those with suspected NVC and percutaneous procedures were used in the absence of NVC. -Personalised, tailored surgical flowcharts were suggested to enhance pain control and reduce recurrence. -MVD provided numerically longer pain-free intervals (61.2 months) than INL (31 months) and MVD/INL together (60 months) when NVC was present, despite no statistically significant difference in pain recurrences (*p* = 0.835).	-Personalised, tailored surgical flowcharts were suggested to enhance pain control and reduce recurrence. -Use of MVD and/or INL in patients with a demyelinating plaque within the trigeminal nucleus or tracts showed marginally significant earlier pain recurrence. -PBC, GKR and RFL were viable alternatives for those with contraindications or absence of NVC but were less durable.
De Nigris Vasconcellos et al., [57]	GKR	Multicentre retrospective cohort study	258/258	-PPMS patients were significantly more likely to have demyelinating lesions at the nerve REZ leading to increased risk of TN during disease course (*p* = 0.018). -Compared to the RRMS cohort, SPMS patients were more likely to have pain controlled and PPMS patients were less likely to have pain controlled at final follow-up (OR 2.0 and 0.389 respectively).	-GKR proved to be a safe and efficient intervention despite a wider range of and more frequent milder complications. -MS subtypes provided potentially important prognostic information with PPMS posing the greatest challenge to pain management.
Sozer et al., [70]	RFA, GKR	Retrospective cohort	37/37	-No statistically significant difference in relapse-free survival between primary GKR and RFA (*p* = 0.906). -Total number of procedures significantly higher in patients with brainstem plaques when GKR used initially (*p* = 0.012). -No significant difference in relapse-free survival after two treatments between patients with and without brainstem plaques (*p* = 0.168).	-RFA and GKR are valid treatment options with similar relapse-free durations. -RFA appears superior for immediate pain relief whilst GKR maintains value with lower incidence of post-operative hypoesthesia.
Bassiouni, Botsch and Zouba, [17]	Arachnoid-CNV Release	Retrospective Cohort	8/37	-75% reported BNI I at final follow-up with pre-operative pain intensity significantly improving post-surgery (*p* < 0.001). -Repeat procedures due to persistent arachnoid strands also delivered pain relief (BNI I).	-Arachnoid-CNV release in medically intractable TN without demonstrable NVC represents a promising emerging option that may be considered prior to ablative procedures, pending further validation in larger cohorts.
Joncas et al., [46]	PSR, MVD, INL	Retrospective Cohort	30/30	-52.3% undergoing solely a PSR required subsequent procedures for pain management. -Patients undergoing a combined regimen of PSR with a MVD and INL had a 75% and 100% BNI I-III respectively at final follow-up. -A combined regimen of PSR with MVD or INL did not increase complication rates.	-PSR may be reserved for patients without NVC or as a rescue/salvage therapy; PSR combined with MVD or INL may provide more durable and effective long-term solutions.
Lange et al., [44]	PSR	Retrospective Cohort	15/48	-60% reported BNI I-II at final follow-up with significant improvement in pain compared to preoperative BNI (*p* < 0.0001). -No significant relation noted between degree of pain relief and extent of rhizotomy.	-PSR is a viable option for refractory or recurrent TN (including MS-TN), especially in the absence of NVC. -MS-TN patients should be informed early about PSR as a treatment option.

Legend: *MVD* microvascular decompression, *GKR* gamma knife radiosurgery, *PBC* percutaneous balloon compression, *GR* glycerol rhizotomy, *RFL* radiofrequency lesioning, *INL* internal neurolysis, *BNI* barrow neurological institute, *mBNI* modified barrow neurological institute, *MS-TN* trigeminal neuralgia secondary to multiple sclerosis, *TN* trigeminal neuralgia, *MS* multiple sclerosis, *SRS* stereotactic radiosurgery, *RRMS* relapsing remitting multiple sclerosis, *PPMS* primary progressive multiple sclerosis, *SPMS* secondary progressive multiple sclerosis, *NVC* neurovascular contact, *MRI* magnetic resonance imaging, trigeminal nerve = cranial nerve 5, *OR* odds ratio, *REZ* root entry zone, *PSR* partial sensory rhizotomy

Percutaneous ablative techniques demonstrated high early efficacy but reduced durability and greater retreatment rates in MS-TN compared with primary TN cohorts [47–49]. GKR yielded comparable initial pain relief but earlier pain recurrence in MS-TN compared to primary TN. Notably, repeat GKR procedures prolonged pain-free intervals. Regarding prognostic factors, multiple studies found that older age and shorter symptom duration prior to GKR predicted favourable outcomes across both primary TN and MS-TN cohorts [50–52] but Zorro et al. [53] found that age over 60 was predictive of greater recurrence. The ranges of reported pain relief and average time to recurrence are reported in Table 4.

### Adverse Outcomes

Adverse effects were generally mild to moderate and procedure-specific. Procedural morbidity was most evident in MVD: Worm et al. [43] found complication rates in MS-TN notably higher than those seen in classical TN, with more severe postoperative sequelae (including a fatality) accompanying MVD compared with percutaneous procedures. PSR was associated with a broad range of neurological complications including hearing and taste impairment, and blurred vision. Trigeminal nerve injury, particularly hypoesthesia, was frequently reported, though rarely bothersome, and anaesthesia dolorosa was noted in 3 cases (7.5%) [44–46].

In contrast, ablative procedures were overall well tolerated, with RFL studies reporting low complication rates with no significant differences between MS-TN and primary TN cohorts [47, 48]. GKR also demonstrated favourable safety profiles with sensory disturbances, particularly trigeminal hypoesthesia, being the most common adverse effects and typically improving over time [50, 54]. Notably, Guillemette et al. [52] found post-procedural hypoesthesia to correlate with longer pain control (*p* < 0.001). Limited data from the included studies on INL suggested minimal complications [55]. Data on arachnoid-CNV release remain limited to a single small study, which reported adverse effects for the overall cohort without stratifying by aetiology. Consequently, it is not possible to determine which, if any, complications occurred specifically in MS-TN patients, and larger prospective studies are required to establish this technique’s safety profile within this population. Overall, while adverse effects were procedure-specific, MS-TN patients particularly tolerated stereotactic interventions well alongside percutaneous interventions. Of all the interventions reported by the included studies, MVD was associated with the highest procedural morbidity Table 4. A full breakdown of adverse effects by modality is described in Table 5.

**Table 4:** Summary of findings from each intervention as well as the reported ranges of pain relief and time to recurrence across all studies

Intervention	Summary of Findings	Range of reported pain relief (BNI I-III) and range of average reported time to recurrence* (TTR) across studies**
MVD	- Efficacy maintained in cases with concurrent NVC. - Limited efficacy noted in absence of NVC. - Diagnostic uncertainty limits conclusions. - Severe complication profile with fatalities noted in wider literature.	Pain Relief: 67–100% Average TTR: 59.4 months***
Percutaneous Ablative Procedures (RFL, GR, PBC)	- Similar early treatment outcomes in MS-TN compared to primary TN cohorts. - Earlier time to recurrence in MS-TN cohorts (limited durability) requiring repeat procedures. - Repeat procedures often equally efficacious. - Mild complication profile.	PBC Pain Relief: 67–100% Average TTR: 8–34 months RFL Pain Relief: 58%-98% Average TTR: 17–60 months GR Pain Relief: 77–100% Average TTR: 17-32.4 months
INL	- Lack of recurrence noted in this small population sample (*n* = 2). - Limited sample size prevents generalisable conclusions.	Pain Relief: 100% Average TTR: N/A
PSR	- May have a role to play in multimodality treatment regimens to prevent recurrence and prolonging pain relief duration. - Severe trigeminal nerve dysfunction (e.g., anaesthesia dolorosa) noted in some cases.	Pain Relief: 95.3–100% Average TTR: 20.1–79 months
GKR	- Mild complication profile. - Similar initial efficacy generally noted in MS-TN cohorts compared to primary TN cohorts. - Repeat procedures are effective in prolonging duration of pain relief. - Limited durability often with dependence on repeat procedures. - Earlier time to recurrence compared to use in primary TN.	Pain Relief: 52-97.3% Average TTR: 7.4–74.5 months
Arachnoid-CNV Release	- Preliminary analysis of Bassiouni, Botsch and Zouba, [17] indicates high efficacy rate (75% BNI I) at final follow-up. - Successful repeat procedures required for persisting arachnoid strands. - Further studies and reviews needed to analyse this relatively new treatment modality – limited dataset limits conclusions.	Pain Relief: 75% Average TTR: NR

Legend: *MVD* microvascular decompression, *GKR* gamma knife radiosurgery, *PBC* percutaneous balloon compression, *GR* glycerol rhizotomy, *RFL* radiofrequency lesioning, *INL* internal neurolysis, *BNI* barrow neurological institute, *MS-TN* trigeminal neuralgia secondary to multiple sclerosis, *TN* trigeminal neuralgia, *MS* multiple sclerosis, *NVC* neurovascular conflict, *MRI* magnetic resonance imaging, Trigeminal Nerve = Cranial Nerve 5, *PSR* partial sensory rhizotomy, *NR* not reported, *NA* not applicable, *TTR* time to recurrence

*Time to recurrence reflects either mean or median values as originally reported by each study; these measures are not statistically interchangeable but are presented together as a descriptive range

**Reported ranges reflect values extracted from included studies for each surgical modality. Given the known heterogeneity across studies in population characteristics, follow-up duration, and outcome definitions, these ranges are presented descriptively; direct comparison between modalities should be interpreted with caution

***Only one study mentioned time to recurrence post-MVD for a complete MS-TN subset, hence the lack of a range

**Table 5:** Table showing the adverse effects noted from each intervention compiled from articles in this present study

			MVD	PBC	GR	RFA	GKR	INL	PSR
Number of Patients			40	138	205	151	629	2	40
**Complication**									
**Trigeminal Nerve Injury**									
	Total Hypoesthesia		2	4	129*	20	92	2	26
		Non-Bothersome		2	11		10		13
		Bothersome				1	71		1
		Unspecified Severity*	2	2	118	19	11	2	12
	Dysesthesia		0	3	0	1	6	0	0
	Paraesthesia		0	0	0	13	9	0	0
	Corneal Abrasion/Reflex Injury		0	0	0	3	4	0	0
	Jaw Weakness		0	2	0	3	4	0	0
	Anaesthesia Dolorosa		0	0	0	0	0	0	3
**Procedural Complication**									
	Meningitis		3	1	3	0	0	0	0
	CSF Leak		3	0	0	0	0	0	1
	Transient Asystole		0	2	0	0	0	0	0
	Balloon Rupture		N/A	1	N/A	N/A	N/A	N/A	N/A
	Buccal Perforation		0	2	0	0	0	0	0
	Pseudomeningocoele		5	0	0	0	0	0	0
	Fatality		1	0	0	0	0	0	0
**Vascular Complications**									
	Intrabuccal Hematoma		0	4	7	4	0	0	0
	Cerebral Venous Sinus Thrombosis		1	0	0	0	0	0	0
	Intracranial Haemorrhage		1	0	0	0	0	0	1
**Other Neurological Complications**									
	Hearing Impairment		2	2	0	0	0	0	1
	Loss of Taste		0	0	0	0	0	0	1
	Blurred Vision		0	0	0	0	0	0	1
	Transient Occipital Neuralgia		0	0	0	0	0	0	1
	Eye Dryness		0	0	0	0	0	0	0
	Herpes Reactivation		0	7	1	0	0	0	1
	Ischemic Stroke		1	0	0	0	0	0	0
	Transient CN III/VI Disruption		0	2	0	0	0	0	0

Legend: *MVD* Microvascular decompression, *GKR* gamma knife radiosurgery, *PBC* percutaneous balloon compression, *GR* glycerol rhizotomy, *RFL* radiofrequency lesioning, *INL* internal neurolysis, *PSR* partial sensory rhizotomy

*The large “unspecified severity” category reflects heterogeneity in original study reporting, where individual studies did not consistently grade findings

### The influence of MS subtype

The included studies in this systematic review presented contrasting findings on the influence of MS subtype on procedural outcomes. Kalluri et al. [56] reported that while MVD provided pain reduction across all MS subtypes, patients with RRMS experienced significantly shorter pain-free intervals compared to those with progressive MS (7.8 vs. 55.6 months; *p* = 0.04) and had higher rates of recurrence (*p* = 0.02). Notably, the degree of post-operative pain relief did not statistically differ between groups [52]. In contrast, De Nigris Vasconcellos et al. [57] in a study of GKR, found that, compared to RRMS patients, patients with SPMS were more likely to achieve pain control (OR 2.0), whereas those with PPMS were less likely (OR 0.389). This study also identified a greater prevalence of demyelinating lesions at the nerve root entry zone in PPMS patients (*p* = 0.018) [57].

### GKR dosages

Nine GKR studies included sufficient data for dose-response analysis [51–54, 57–61] Table 6. Across eight studies, no statistically significant association was identified between higher radiation dose and improved pain outcomes. However, one study, Mathieu et al., [61], found that doses > 80 Gy were associated with an increased likelihood of pain relief (*p* = 0.003) and lower risk of pain recurrence (*p* = 0.002). GKR dosages, target locations and corresponding outcomes are summarised in Table 6.

**Table 6:** Table showing the different GKR dosages and target locations in articles investigating GKR and their resulting outcomes

Author and Year	GKR Dosages and Treatment Location	Outcome
Zorro et al., [53]	Median Maximum Radiation Dose (Gy): 80 (range: 70–90) Described Treatment Location: 2–8 mm anterior to the junction of trigeminal nerve and pons Isocentre Size: Single 4-mm isocentre	Initial Success (BNI I-III) Rate: 97.2% Recurrence Rate: 37.8% Median Time to Pain Recurrence (months): 74.5 (95% CI: 39.7-109.3) (range 3–80) Final Success (BNI I-III) Rate: 62.1% Statistical Conclusion: Following multivariate analysis, maximum dose was not predictive of pain recurrence after GKR.
Mathieu et al., [61]	Maximum Radiation Dose (Gy): 80–90 - 80 Gy (n = 19) - 85 Gy (n = 7) - 90 Gy (n = 1) Described Treatment Location: Cisternal segment of the trigeminal nerve Isocentre Size: Single 4-mm isocentre	Initial Success (BNI I-III) Rate: 81.5% Recurrence Rate: 51.9% Median Time to Pain Recurrence (months): 26.5 Statistical Conclusion: Cox regression analyses demonstrated that doses higher than 80 Gy were associated with an increased likelihood of pain relief (p = 0.003) and lower rates of pain recurrence (p = 0.002).
Weller et al., [59]	Median Maximum Radiation Dose (Gy): 90 (range: 80–90) Described Treatment Location: A variety of targeting philosophies were used from targets close to the dorsal root emergence to further from the brainstem including the pars triangularis. Targets include: - the dorsal root emergence (DRE) - pons - distal nerve at the point where it pierced the petrous dura Isocentre Size: Single 4-mm isocentre	Initial Success (BNI I-III) Rate: 82% Recurrence Rate: 40.7% Median Time to Pain Recurrence (months): 9.6 (range: 1.3-3) Final Success (BNI I-III) Rate: 48.4% Statistical Conclusion: Following multivariate analysis, none of the dosimetry factors (DRE dose, pons maximum dose, prescription dose or nerve length) predicted efficacy of treatment.
Przybylowski et al., [60]	Median Maximum Radiation Dose (Gy): 85 (range: 50–85) Described Treatment Location: Root entry zone Isocentre Size: Single 4-mm isocentre	Initial Success (BNI I-III) Rate: 26.2% Median Time to Pain Recurrence (months): 16 (range: 1–84) Statistical Conclusion: Following multivariate analysis, treatment dose was not predictive of pain control after treatment.
Xu et al., [58]	Median Maximum Radiation Dose (Gy): 80 (range: 40–90) Described Treatment Location: 2–8 mm anterior to the junction of trigeminal nerve and pons Isocentre Size: Single 4-mm isocentre	Initial Success (BNI I) Rate: 29.3% Median Time to Pain Recurrence (months): 14.1 (range: 0.33–120) Statistical Conclusion: For all patients, maximum radiation dosage did not predict initial pain outcomes or maintenance of pain-free intervals.
Guillemette et al., [52]	Median Maximum Radiation Dose (Gy): 80 (range: 70-88.9) Described Treatment Location: Cisternal segment of trigeminal nerve Isocentre Size: Not specified	Initial Success (BNI I-III) Rate: 81.8% Recurrence Rate: 44.4% Median Time to Pain Recurrence (months): 8.3 (range: 0.6–85.0) Final Success (BNI I-III) Rate: 45.5% Statistical Conclusion: A univariate Cox survival analysis showed no statistically significant difference on maximum pain dosage < 80 or > 80 Gy.
Leduc et al., 2023 [50]	Median Maximum Dose (Gy): 80 (range 60–90) Median Treatment Duration (minutes): 40 (range: 28–63) Described Treatment Location: Cisternal segment of trigeminal nerve root Location in Repeat GKR: Moved distally in cisternal segment Isocentre Size: Single 4-mm isocentre	Initial Success (BNI I-III) Rate: 76.7% Median Time to Pain Recurrence (months): 26 Statistical Conclusion: Following a Cox regression analysis, treatment dosage was not associated with recurrence.
Öztürk Özlük et al., [54]	Median Maximum Radiation Dose (Gy): 80 (range: 80–90) Described Treatment Location: Anterior cisternal segment of trigeminal nerve Isocentre Size: Single 4-mm isocentre	Initial Success (BNI I-III) Rate: 71.4% Recurrence Rate: 30% Median Time to Pain Recurrence (months): 35 (range: 12–180) Final Success (BNI I-III) Rate: 50% Statistical Conclusion: The size of the cohort hindered the study’s ability to perform statistical analyses on applied dosages.
Franzini et al., [51]	Median Maximum Dose for Repeat GKR (Gy): 85 (IQR: 75–80) Described Treatment Location: Cisternal segment of trigeminal nerve Median Distance from trigeminal nerve REZ (mm): 6.65 (IQR 5.2–7.9) Distal/Proximal to Primary Target: 14/4 patients Isocentre Size: Single 4-mm isocentre	Initial Success (BNI I-III) Rate: 83.3% Recurrence Rate: 33.3% Median Time to Pain Recurrence (months): 12 (IQR: 3–12) Final Success (BNI I-III) Rate: 55.6% Statistical Conclusion: For all patients, maximum radiation dosage did not predict pain reduction.
De Nigris Vasconcellos et al., [57]	Median Maximum Radiation Dose (Gy): 80 (range 70–90) Median Treatment Duration (minutes): 44.14 (range: 28.14–95.2) Described Treatment Location: 2–8 mm anterior to the junction of trigeminal nerve and pons Isocentre Size: Single 4-mm isocentre	Initial Success (BNI I-III) Rate: 84.6% Median Time to Pain Recurrence (months): 10 Statistical Conclusion: A subgroup analysis for maximum dosage (< 80 Gy vs. > 80.1 Gy) showed no significant difference in pain outcomes observed.

Legend: Trigeminal nerve = Cranial Nerve 5, *BNI* barrow neurological institute, *REZ* root entry zone, *IQR* interquartile range

## Discussion

### Overview

The results from our systematic review indicated that all major surgical interventions, including percutaneous ablative techniques, MVD and GKR, provided high rates of initial pain relief (BNI I-III) for MS-TN with comparable initial outcomes to those found in primary TN. However, the durability of pain relief was significantly poorer in MS-TN with percutaneous modalities particularly demonstrating shorter pain-free periods, higher recurrence rates and a greater consideration of repeat procedures in that patient cohort. MVD and PSR, whilst altogether providing a more sustained benefit than percutaneous alternatives, was associated with more severe complications. In contrast, percutaneous techniques and GKR offer more favourable safety profiles with complications typically being mild. Factors influencing outcomes included the patient’s underlying neuroanatomical/inflammatory profile, MS subtype (with some evidence suggesting differential results in relapsing-remitting forms compared to progressive forms), and patient age rather than radiation dosage in the case of GKR. Overall, the findings underscored that whilst surgical management can be effective for initial symptom control in MS-TN, the disease’s underlying pathophysiology leads to less sustained benefit than in classical TN. This necessitates a tailored, and potentially multi-procedural long-term treatment strategy.

### Microvascular decompression and the influence of neurovascular conflict and demyelinating plaques

Analysis of the included studies suggests that MVD is less effective and associated with higher complication rates in MS-TN than in primary TN. The study by Worm et al. [43] reported that only 2/7 MS-TN patients achieved BNI I outcomes at final follow-up, with one fatality. The study by Mazzapicchi and colleagues [71] demonstrated longer pain-free intervals following MVD when NVC was present (outperforming percutaneous alternatives), but earlier recurrence when trigeminal demyelinating plaques were also identified. This supports the hypothesis that demyelinating plaques worsen outcomes [4]. Notably, the remaining 5/7 MVD patients in Worm et al. [43] had demyelinating plaques without trigeminal nerve morphological changes, suggesting a reduced pathological role of NVC in MS-TN patients.

These pain outcomes in the presence of NVC in MS-TN cohort must be interpreted in the context of diagnostic uncertainty. Some studies included in this review such as Kalluri et al., [56], Paulo et al., [15] reported that a large proportion of their MS-TN cohort had NVC. However, other studies did not explicitly stratify the MVD MS-TN cohort by either the presence or absence of plaques or NVC. As such, it is not possible to confidently determine whether TN in those study participants was a direct consequence of their MS demyelinating pathology or whether their MS was an incidental co-morbidity. This fundamental uncertainty limits the interpretability of MVD outcomes in the true MS-TN population, introducing significant selection bias. MVD outcomes may be thus biased toward those expected in classical TN and thus not generalisable to MS-TN cohorts.

The role of concurrent NVC in MS-TN remains debated. Noory et al. [72] identified a strong association between ipsilateral symptomatic demyelinating plaques on the affected side and a low prevalence of NVC with morphological changes. This contradicts Truini et al. [73] which proposed a “double-crush” mechanism involving concurrent NVC with morphological changes and demyelinating brainstem plaques. Both studies used similar MRI protocols with powerful 3T MRI, but Noory et al. [72] recruited and included more patients from a tertiary headache centre versus an MS-specific cohort in Truini et al. [73]. Noory et al. [72] still acknowledged MVD’s role in the small subset with NVC and morphological changes.

A key challenge is that MRI may not reliably distinguish nerve morphological changes secondary to vascular conflict from those caused by demyelinating plaques [74, 56]. However, Truini et al. [73] reported high diagnostic specificity for NVC using predefined MRI criteria assessed by expert neuroradiologists. Advanced MRI techniques, including diffusion tensor imaging, may further aid differentiation by identifying trigeminal nerve microstructural integrity changes associated with plaque-related injury [75].

Data from Worm et al. [43] implied that MVD retained some (albeit reduced) effectiveness in providing pain relief in an MS-TN cohort, supporting the possibility of concurrent NVC in the pathophysiology for that cohort. Paulo et al. [15] recommended detailed pre-operative MRI, suggesting that the absence of plaques near the trigeminal nucleus may indicate NVC-driven pain, making MVD viable. However, Broggi et al. [76] reported only 60% accurate pre-operative NVC MRI diagnoses, with 17% experiencing false negatives, though MRI advances in the 16-year gap between publications may be a relevant factor in this discrepancy. For example, Broggi et al. [76] used only 0.5-1.5T echo T2-weighted MRI scanning compared to the more intricate FIESTA MRI sequences in Paulo et al. [15]. FIESTA MRI sequences allow for suppression of background tissues and thus can provide effective contrast for anatomical visualisation of small structures and individual nerves [77].

Whilst high-resolution preoperative imaging is important in personalised management, the presence of NVC may simply allow for a greater consideration of MVD as an option within a patient’s individualised care pathway. Conversely, the absence of NVC on imaging may support consideration of alternative treatment approaches, as the likelihood of benefit from MVD would then be substantially lower.

### Gamma knife radiosurgery

Across the studies included in this review, GKR provided similar initial pain relief in refractory MS-TN (where the pain had not responded to standard medical therapy) compared with classical or idiopathic TN. For example, Leduc et al. [50] reported initial pain control rates of 77% in MS-TN and 69% in controls (classical/idiopathic TN) respectively. Öztürk Özlük et al. [54] found a similar initial pain relief rate (71.4%) in MS-TN patients with prior failed treatments, highlighting its value in resistant cases. The role of GKR in a MS-TN management pathway was further emphasised in Sozer et al., [69]. Sozer et al., [70] demonstrated that in patients who had already undergone two prior treatments, there was no significant difference in relapse-free survival post-GKR between patients with or without brainstem plaques. Whilst that finding derives from a single included study with prospective validation undoubtedly necessary, case series such as Conti et al., [78] also found that brainstem plaques were not predictive of GKR failure. Guillemette et al. [52], De Nigris Vasconcellos et al. [57] and Rashid et al., [68] further supported GKR’s efficacy in medically refractory cases, emphasising minimal incapacitating complications. These initial pain control figures closely align with prior systematic reviews (Ali et al. [25]: 83.3%; Helis et al. [79]: 82%), reinforcing GKR as a typically well tolerated, effective option in intractable MS-TN.

The influence of patient age has been inconsistently reported across studies. Some studies determined older age as a consistent predictor of more sustained responses [51, 52], consistent with earlier small-sample studies by Han et al. [80] and Karam et al. [81]. This potentially reflects more prominent inflammatory, clinical and subclinical activity with more frequent MS relapses in younger individuals [82]. However, other included studies determined age at the time of GKR as non-predictive of pain outcomes; one included study – Zorro et al., [53] - found older age to be associated with greater recurrence. These discrepancies may be due to patient heterogeneity. The opposing stances within the literature (despite an overall preponderance toward better outcomes in older patients) suggest that age alone should not drive treatment decisions.

Both this current systematic review and prior literature highlight limited GKR durability in MS-TN. Guillemette et al. [52], Leduc et al. [50] and Verheul et al., [69] reported longer-lasting relief in primary TN, with MS-TN patients experiencing higher recurrence rates. Recurrence rates in the wider literature vary widely (0-52.2%) due to differences in protocols, follow-up, and patient heterogeneity [25]. While recurrence may reflect MS-TN’s pathophysiology or MS progression itself [80], repeat GKR can extend pain relief with good tolerability [28, 51, 69], though repeated procedures may compromise patient convenience.

### Percutaneous ablative procedures

The studies included in this review support the efficacy and relative tolerability of percutaneous procedures (PBC, RFL, GR). Worm et al. [43], Gündüz et al. [48], Lozouet et al. [49], Staudt et al., [24] reported similar initial effectiveness of percutaneous ablative procedures in MS-TN and primary TN, despite more frequent recurrences in the MS-TN cohort. In the wider literature, reported recurrence rates varied widely (Alvarez-Pinzon et al. [83]: 50%; Martin, Teo and Suttner [84]. : 85%), reflecting differing sample sizes, follow-up duration and patient heterogeneity.

Surgical variables - like thermocoagulation temperature and balloon compression duration - were inconsistently reported [85]. While a pear-shaped balloon predicts better durability, as seen in Montano et al., [67], its subjective role as a visual proxy for catheter positioning and compression underscores the importance of surgical expertise [86, 87]. Different percutaneous techniques offer further practical advantages. For example, RFL can be performed on a day-case basis, and Mousavi, Gehling and Burchiel [47] reported no statistically significant outcome differences between primary and repeat procedures.

### Internal neurolysis

While the total INL sample size from the included studies in this current systematic review (*n* = 2) severely limits generalisability, other studies support its efficacy. Durnford et al. [55] reported favourable outcomes in patients with an MRI-confirmed absence of NVC prior to surgery. This is consistent with previous suggestions that INL may provide more durable relief and greater efficacy than other percutaneous alternatives [88]. The absence of recurrence in these cases implies that MS-TN is not invariably driven by focal pontine demyelination (INL does not target these lesions), although the precise mechanism of INL remains unclear [4, 55].

### Arachnoid-trigeminal nerve release

This review enabled a preliminary assessment of a novel microsurgical intervention in MS-TN involving release of the trigeminal nerve from the ensheathing arachnoid. Notably, in the single study analysed, all MS patients exhibited a typical arachnoid-CNV conflict indistinguishable from that seen in the primary TN cohort, despite an absence of classical NVC [17]. Demyelinating plaques were identified in only half of the MS cohort. This raises the possibility that arachnoid-CNV conflict, rather than demyelination alone, may play a pivotal role in TN pathogenesis in the absence of NVC [17]. This finding should be interpreted cautiously, however, given the small sample size and lack of blinded pathological correlation. The achievement of complete pain relief with no need for medication (BNI I) in 75% of patients supports the notion that direct mechanical decompression may address the underlying pathology [17].

This study lacked MS-TN-specific data regarding adverse outcomes; however, in the wider TN cohort (MS and non-MS related, *n* = 37), adverse effects were limited to transient dizziness (*n* = 4), hypoacusis (*n* = 4), diplopia (*n* = 3), and facial hypoesthesia (*n* = 2) [17]. While these findings support consideration of non-ablative microsurgical release for medically intractable TN without NVC, the greater invasiveness of open surgery relative to percutaneous procedures warrants careful patient selection, and further analysis of the pathophysiology of MS-TN remains important to optimising management pathways [17].

### Partial sensory rhizotomy

The studies included in this current systematic review support both the efficacy and durability of PSR in refractory cases of MS-TN, particularly in the absence of NVC. This is consistent with a lack of vascular conflict in MS-TN aetiology. Bigder et al., [45] and Lange et al. [44] suggest that PSR should be utilised earlier in the disease course of refractory MS-TN patients to reduce reliance on percutaneous procedures associated with inferior pain outcomes in this cohort. One of the three articles focusing on PSR in this current systematic review – Joncas et al., [46] - reported only a 33% pain free rate at final follow-up. Notably, high-resolution preoperative MRI was only available prior to half of the procedures, precluding a robust analysis of imaging findings [46]. This is clinically relevant as the aetiology of TN may be due to vascular conflict and hence the findings may not be truly representative of a MS-TN cohort. This interpretation is further supported due to the 100% and 75% initial and final pain relief rate when MVD was used alongside PSR in this study. This may also further highlight the potential role PSR may play in multimodality treatment when NVC is present. The wider literature further supports the excellent efficacy and durability of PSR in MS-TN cohorts with Li et al., [89] reporting a 100% and 81% response rate at initial and final follow up. This study corroborates Bigder et al.,’s [45] findings that PSR was associated with a significantly lower pain score (*p* < 0.05) and significantly lower recurrence rate than PBC at all points of follow-up post-surgery (*p* < 0.05).

### Gamma knife radiosurgery dosages

Ten studies included in this current systematic review reported GKR dosages ranging from 40 to 90 Gy, with six using median maximum doses of approximately 80 Gy. Across eight studies, no statistically significant association was identified between radiation dose and pain outcomes or recurrence [50, 51, 52, 54, 57]; one study lacked statistical power and one study found that doses > 80 Gy correlated with better outcomes. Mathieu et al., [61] who did find a statistically significant relationship between dosage and better outcomes had the smallest sample size out of all the GKR studies analysed, potentially contributing for their differing conclusion. The general preponderance toward a lack of association between dose and pain outcomes is consistent with prior MS-TN literature [79, 60], though small sample size and follow-up bias reduced confidence in these results.

This contrasts with primary TN literature, where higher doses (> 90 Gy vs.<82 Gy) yielded more durable pain relief [90–92]. The discrepancy may reflect MS-TN’s more complex inflammatory and neurodegenerative pathophysiology, suggesting that dose escalation provides limited additional benefit while potentially increasing toxicity. Furthermore, high radiation doses in areas with ongoing autoimmune demyelination could exacerbate autoimmune damage and toxicity [79].

### Multiple sclerosis subtypes

The studies included in this current systematic review report conflicting findings on the influence of different underlying MS subtype on MS-TN prognosis. Kalluri et al. [56] reported worse long-term TN outcomes in RRMS patients, with a higher pain recurrence likelihood at 36 months, though MS subtype did not predict final pain severity. This may be due to the more intense systemic and intrathecal inflammation seen in RRMS [93]. In contrast, De Nigris Vasconcellos et al. [57] with a larger cohort, found that the subtype did predict recurrence (*p* = 0.045), with PPMS having a shorter median time to recurrence (9.8 vs. 11.1 months in RRMS) and poorer final pain control.

Evidence for how MS-TN responds to different surgical interventions based on MS subtype remains limited. Available data, however, suggest a critical distinction. Kalluri et al. [56] found MVD to have worse outcomes specifically in patients with RRMS, while De Nigris Vasconcellos et al. [57] reported that GKR provided less benefit in progressive forms of MS, particularly PPMS. The reduced efficacy of MVD - a treatment designed for vascular conflict - in RRMS challenges a purely compressive aetiology for MS-TN. This finding aligns with the hypothesis that TN in RRMS may instead be driven by a heightened central neuroinflammatory profile [94]. This mechanistic link is further supported by the epidemiological observation that TN occurs primarily in RRMS patients [95].

Kalluri et al., [56] reported that 96% of their studies MS-TN cohort had NVC. However, some of those TN cases may have been related to demyelinating MS pathology either in addition to, or instead of, NVC. Moreover, despite the presence of a diagnostic framework, the pathophysiological boundaries of MS phenotypes are often unclear [96]. Stratification of patients into subtypes is often based on subjective clinical assessment and transition between subtypes is challenging to ascertain. The use of biomarkers may aid in the differentiation of MS subtypes and thus treatment regimens.

### Role of biomarkers

Recent exploratory research by Lafta et al. [97]. identified increased serum and CSF levels of specific proteins (SFRP1, FKBP5, TBCB) in classical TN patients compared to both MS patients and controls, with successful MVD normalising these levels postoperatively. These proteins have been implicated in neuroinflammation, chronic stress response, and neurodegeneration respectively, potentially offering novel insight into TN pathophysiology. However, these findings should be interpreted cautiously given the small sample size, particularly regarding postoperative CSF normalisation which was demonstrated in only eight patients. Whether similar biomarker profiles exist in MS-TN patients, or whether protein normalisation occurs following other surgical modalities, remains unknown. Further research examining these markers specifically in MS-TN cohorts across different treatment modalities may help inform surgical decision-making and contribute to the development of a personalised treatment algorithm.

### Adverse effects

Table 5 summarises intervention-specific complications from the twenty-two studies that included detailed breakdowns by procedure. Across GKR, PBC, INL, RFA, PSR and GR hypoesthesia was the most frequent adverse effect, though it was infrequently noted as being bothersome. Of note, different studies classified complications differently. Some studies classified non-bothersome hypoesthesia as an expected surgical consequence rather than a complication while others grouped all hypoesthesia together as complications [70, 24, 68]. This heterogeneity complicates direct comparison of hypoesthesia rates across procedures and underscores the need for standardised complication reporting.

Hypoesthesia related to trigeminal nerve sensory dysfunction from PSR or GR may conceivably be further amplified in MS-TN cohorts with inflammation-induced prolonged trigeminal nerve fragility and vulnerability [54]. Both INL patients developed hypoesthesia; previously reported rates of 20–96% [88, 98, 99] likely reflect prior patient variability, and procedural differences. GR-related hypoesthesia, usually transient but more common after repeat procedures, matched previously published rates of 23.3–72% [18, 100]. Some studies analysed in this review further suggest a potential link between post-intervention hypoesthesia and pain relief; Guillemette et al. [52] proposed that treating patients to the threshold of clinical numbness could predict GKR efficacy. This hypothesis may shift treatment paradigms from solely minimising numbness to maximising pain control [101].

Table 5 links RFL to masticatory weakness, dysesthesia, and corneal damage. This may be related to significant individual variation of somatotropic organisation of trigeminal nerve fibres and the irreversible damage to small, unmyelinated nociceptive fibres [102, 103]. Curved electrodes may reduce sensory complications through more selective lesioning [18]. Higher balloon pressures and repeat PBC increase risks, including trigeminal nerve injury. Misplacement of the balloon can damage other cranial nerves, with the abducens nerve being the most commonly damaged, leading to diplopia [18, 104].

Percutaneous procedures featured a broader but generally milder range of side effects, compared to MVD and PSR.

PSR, which is ultimately a destructive procedure, is strongly associated with hypoesthesia post-surgery although, the studies included in this current systematic review indicate that this seldom becomes bothersome [44–46]. Whilst only noted in a minority of PSR patients in this current systematic review, severe trigeminal nerve dysfunction such as anaesthesia dolorosa should be considered when counselling patients and planning surgery [44]. The proportion and location of sensory root undergoing transection in PSR may relate to surgical efficacy and post-operative complications. Whilst expanding the range of sensory root transection may reduce post-operative recurrence, it may increase the risk of broader neurological dysfunction [16, 105]. Further, cutting the sensory root as close to the pons as possible may further reduce incidence of facial numbness [16, 106]. Historically, the higher complication post-PSR has been cited against its use; two of three studies analysing PSR in this current systematic review encompassing 24 cases did not find any major operative or neurological complications [44, 45]. With direct visualisation allowing for precise tailoring of fibre sectioning based on pain distribution and prospective studies analysing the optimal treatment location, PSR may play an important role in MS-TN management pathways [44].

One surgery-related fatality post-MVD was reported in Worm et al. [43] also described in an earlier larger case series e.g., Barker *et* al. [107]. Hearing impairment after MVD is reported to be as high as 10% in some studies [108]. Vascular complications, including ischaemic stroke, are known MVD risks [109].

### Demographics

Across included studies (Table 2), MS-TN showed a clear female predominance, reflecting known MS epidemiology and mirroring gender distributions seen in classical TN. Females have an association with smaller posterior fossae (a hypothesised TN risk factor), greater odds of reporting TN, and are more likely to develop MS and experience relapses [13, 110–115].

As highlighted in Table 2, several included studies found that MS-TN presented at a significantly younger age than classical TN [48–50]. This reflects Maarbjerg et al. [116]’s epidemiological review which compared classical TN’s average age at onset of 53 with secondary TN’s average age at onset of 43. Whilst younger age increases the likelihood of secondary TN, its absence does not rule MS-TN out, as highlighted by the overlapping age ranges between primary TN and MS-TN cohorts [4].

Notably, none of the included studies reported patient ethnicity, highlighting an important gap in current MS-TN research. Identified studies in this review were predominantly from Western Europe and North America, limiting generalisability to high-income resource-rich health systems. Whilst MS, TN and consequently MS-TN appear less prevalent in South America, Africa and Asia [117–119], epidemiological data from low-income countries remains sparse [118]. Notably, dramatic rises in MS prevalence in countries such as Egypt and Algeria may reflect improvements in diagnostic technology rather than genuinely increasing incidence [119]. Substantial disparities in neurosurgical trial publications between high- and low-income countries have been documented [120]. This is attributed to limited financial resources, inadequate infrastructure, and neurosurgeon scarcity [121] and compounded by an absence of standardised neurosurgical guidelines [122]. Systematic reviews of this nature represent an important step toward establishing standardised international guidelines for MS-TN surgical management. This may ultimately help bridge the disparity in neurosurgical care access between high- and lower-income settings. It is to be hoped that future studies on surgical interventions for MS-TN will include a more representative spread of countries to help inform such guidelines.

### Key considerations and limitations

The studies identified in this systematic review showed a wide variability in follow-up duration, enrolment criteria, and timing between MS diagnosis and TN onset (as shown in Table 2). This limits comparisons as short follow-ups may underreport complications or recurrences, particularly for interventions like GKR with delayed effects [123]. Early-onset MS-TN can precede or coincide with MS onset, whereas later-onset TN with advanced MS, as described by Ferraro et al. [124] is linked to greater disability (measured using the EDSS scale) and more frequent surgical intervention. This likely reflects reduced tolerability from more advanced/irreversible demyelination associated with natural MS progression; thus, TN onset in earlier stages of disability may correlate with inflammatory/reversible mechanisms rather than degenerative/irreversible ones [123, 125]. Another important limitation of this review is the diagnostic uncertainty inherent in MS-TN where NVC is present. In such cases, vascular conflict may represent the primary pain driver, consistent with a “double-crush” mechanism, or MS may represent an incidental co-morbidity of such patients rather than a direct contributor to pain aetiology. Moreover, heterogeneity in complication reporting limits the validity of comparisons across treatment modalities as studies varied in their classification of expected surgical sequelae, severity grading, and follow-up duration over which complications were recorded. Quality of life was not formally analysed in the studies identified in this systematic review and future research should directly cover this important aspect. A further limitation is the restriction to English-language studies, which may have excluded relevant data published elsewhere and introduces the possibility of language-related selection bias.

Furthermore, the uneven distribution of patient numbers across modalities may limit comparability within this current systematic review. This imbalance likely reflects both temporal publication trends and GKR’s safe, non-invasive nature which facilitates larger cohort sizes and procedural volumes. This is seen in Xu et al., [58] and De Nigris Vasconcellos et al., [57] which contributed 263 and 258 GKR patients respectively, with these two articles together comprising more than half of the total GKR population analysed in this review.

Following our NOS assessment for risk of bias (see Supplementary Tables 1 and 2), eight articles were of moderate quality, and twenty-four articles were of good quality. The main limitations found through this assessment were related to comparability and selection domains; most studies were retrospective, increasing susceptibility to selection bias, and comparability across cohorts was limited. The lack of a low-quality subset of articles further supported the decision to not pursue exclusion-based sensitivity testing.

Although studies from the year 2000 onwards were eligible for inclusion, the earliest included study was published in 2009. This may reflect the natural growth in MS-TN surgical literature in the modern diagnostic era following standardisation of MS diagnostic criteria. However, given the 17-year span between the oldest and most recent included studies, diagnostic criteria may still have varied across the included studies as the McDonald Criteria underwent substantial revisions in 2010 and 2017, each introducing meaningful changes to MS diagnostic standards.

## Future directions and conclusions

This study highlights evidence that MS-TN should be considered as a disease category in its own rights based on surgical treatment outcomes. The data in this systematic review indicate a consistent pattern: percutaneous and stereotactic approaches appear safer and provide comparable short-term pain relief to MVD in MS-TN, but relief is less durable and retreatment is more frequent. All procedures show initial benefit, yet recurrence, durability, and complication rates differ by intervention. Outcomes are influenced by key factors such as NVC, patient age, and prior treatments. Current literature on MS-TN surgical management is limited by small cohorts and inconsistent reporting, highlighting the need for standardized follow-up and BNI scoring. Future studies should examine MS subtypes, MS-TN pathophysiology, and intervention-specific variables to optimize outcomes. Cohort multiple RCTs could evaluate combined treatment strategies integrating surgery with pharmacotherapy or adjuncts. Preoperative neuroanatomical assessment, particularly MRI with 3D FIESTA, may improve patient selection by characterizing trigeminal nerve morphology and NVC. Personalized treatment algorithms incorporating age, prior therapy, plaques, and NVC may streamline decision-making, potentially reduce unnecessary procedures, and may help improve quality of life in MS-TN. The development of formal evidence-based guidelines specific to MS-TN would be an important step to help guide management in this vulnerable patient cohort.

Not Applicable.

## Supplementary Information

Below is the link to the electronic supplementary material.

Supplementary Material 1

## Data Availability

All data generated or analysed during this study are included in this published article [and in supplementary information files] and fully referenced accordingly.

## Abbreviations

MS: Multiple sclerosis
TN: Trigeminal neuralgia
MS: TN–Trigeminal neuralgia secondary to multiple sclerosis
GKR: Gamma knife radiosurgery
MVD: Microvascular decompression
INL: Internal neurolysis
RFL: Radiofrequency lesioning
PBC: Percutaneous balloon compression
GR: Glycerol rhizotomy
CNV: Cranial nerve 5 (trigeminal nerve)
RRMS: Relapsing remitting multiple sclerosis
PPMS: Primary progressive multiple sclerosis
SPMS: Secondary progressive multiple sclerosis
BNI: Barrow neurological institute
NVC: Neurovascular contact
PSR: Partial sensory rhizotomy
