# Supplementary material for: The efficacy of surgical interventions for trigeminal neuralgia in multiple sclerosis: A systematic review
Source: Neurosurg Rev. 2026 Oct 7;49(1):600. doi: 10.1007/s10143-026-04494-5 (PMC13642504; doi:10.1007/s10143-026-04494-5)
Supplement: Supplementary file 1 [file 10143_2026_4494_MOESM1_ESM.docx]

Supplementary Table 1: Risk of Bias Assessment (NOS) for included cohort studies

| **Author and Year - Cohort Studie(s)** | **Selection** | | | | **Comparability** | **Outcome** | | | **Overall** | **Category** |
| --- | --- | --- | --- | --- | --- | --- | --- | --- | --- | --- |
|  | **Representativeness of the Exposed Cohort** | **Selection of the Non-Exposed Cohort** | **Ascertainment of Exposure** | **Outcome of Interest** |  | **Assessment of Outcome** | **Follow-Up Period Duration** | **Cohort Size at Final Follow-Up** |  |  |
| Worm et al., 2023 | * |  | * | * |  | * | * | * | 6 | **Moderate** |
| Mousavi et al., 2022 | * |  | * | * | * | * | * | * | 7 | **Good** |
| Gündüz et al., 2023 | * | * | * |  |  | * | * | * | 6 | **Moderate** |
| Öztürk Özlük et al., 2024 | * |  | * | * | * | * | * | * | 7 | **Good** |
| Lozouet et al., 2024 | * | * | * |  | * | * | * | * | 7 | **Good** |
| Franzini et al., 2024 | * |  | * | * | ** | * | * | * | 8 | **Good** |
| Kalluri et al., 2023 | * |  | * |  | ** | * | * | * | 7 | **Good** |
| Mazzapicchi et al., 2024 | * |  | * | * | * | * | * |  | 6 | **Moderate** |
| De Nigris Vasconcellos et al., 2024 | * |  | * | * | * | * | * | * | 7 | **Good** |
| Guillemette et al., 2022 | * | * | * |  | ** | * | * | * | 8 | **Good** |
| Durnford et al., 2022 | * | * | * |  |  | * | * | * | 6 | **Moderate** |
| Asplund et al., 2019 | * |  | * |  | * | * | * | * | 6 | **Moderate** |
| Krishnan et al., 2017 | * |  | * | * | ** | * | * | * | 8 | **Good** |
| Lee et al., 2020 | * |  | * |  | * | * | * |  | 5 | **Moderate** |
| Xu et al., 2019 | * |  | * |  | ** | * | * | * | 7 | **Good** |
| Noorani et al., 2019 | * | * | * |  | ** | * | * | * | 8 | **Good** |
| Tuleasca et al., 2014 | * |  | * |  | ** | * | * | * | 7 | **Good** |
| Weller et al., 2014 | * |  | * | * | ** | * | * | * | 8 | **Good** |
| Montano et al., 2012 | * |  | * |  | ** | * | * | * | 7 | **Good** |
| Rashid et al., 2018 | * | * | * |  | ** | * | * | * | 8 | **Good** |
| Staudt et al., 2019 | * | * | * |  | ** | * | * | * | 8 | **Good** |
| Bigder et al., 2019 | * | * | * |  | * | * | * | * | 6 | **Moderate** |
| Paulo et al., 2019 | * |  | * | * | ** | * | * | * | 8 | **Good** |
| Przybylowski et al., 2018 | * |  | * |  | ** | * | * | * | 7 | **Good** |
| Zorro et al., 2009 | * |  | * |  | ** | * | * | * | 7 | **Good** |
| Mathieu et al., 2012 | * |  | * |  | ** | * | * | * | 7 | **Good** |
| Verheul et al., 2010 | * | * | * |  | ** | * | * | * | 8 | **Good** |
| Sozer et al., 2025 | * |  | * |  | ** | * | * | * | 7 | **Good** |
| Joncas et al., 2026 | * |  | * |  | * | * | * | * | 6 | **Moderate** |
| Lange et al., 2026 | * | * | * | * | * | * | * | * | 8 | **Good** |
| Bassiouni, Botsch and Zouba, 2025 | * | * | * | * | * | * | * | * | 8 | **Good** |

Supplementary Table 2: Risk of Bias Assessment (NOS) for included case-control studies

| **Author and Year - Case Control Studies** | **Selection** | | | | **Comparability** | **Exposure** | | |  |  |
| --- | --- | --- | --- | --- | --- | --- | --- | --- | --- | --- |
|  | Case Definition | Representativeness | Selection of Controls | Definition of Controls |  | Ascertainment of Exposure | Method of Ascertainment | Non-Response Rate | Overall | **Category** |
| Leduc et al., 2023 | * | * |  | * | ** | * | * | * | 8 | **High** |
